# Genetic evidence for male-biased dispersal in the Qinghai toad-headed agamid *Phrynocephalus vlangalii* and its potential link to individual social interactions

**DOI:** 10.1002/ece3.532

**Published:** 2013-03-20

**Authors:** Yin Qi, Weizhao Yang, Bin Lu, Jinzhong Fu

**Affiliations:** 1 Chengdu Institute of Biology, Chinese Academy of SciencesChengdu, Sichuan, 610041, China; 2 Department of Integrative Biology, University of GuelphGuelph, Ontario, N1G 2W1, Canada

**Keywords:** Lizards, mating system, microsatellite DNA, morphology, *Phrynocephalus vlangalii*, population genetic structure, sex-biased dispersal, social interaction

## Abstract

Sex-biased dispersal has profound impacts on a species' biology and several factors have been attributed to its evolution, including mating system, inbreeding avoidance, and social complexity. Sex-biased dispersal and its potential link to individual social interactions were examined in the Qinghai toad-headed agamid (*Phrynocephalus vlangalii*). We first determined the pattern of sex-biased dispersal using population genetic methods. A total of 345 specimens from 32 sites in the Qaidam Basin were collected and genotyped for nine microsatellite DNA loci. Both individual-based assignment tests and allele frequency-based analyses were conducted. Females revealed much more genetic structure than males and all results were consistent with male-biased dispersal. First-generation migrants were also identified by genetic data. We then examined eight social interaction-related morphological traits and explored their potential link to sex-biased dispersal. Female residents had larger heads and longer tails than female migrants. The well-developed signal system among females, coupled with viviparity, might make remaining on natal sites beneficial, and hence promote female philopatry. Dominant females with larger heads were more likely to stay. Contrary to females, male migrants had larger heads and belly patches than residents, suggesting that dispersal might confer selective advantages for males. Such advantages may include opportunities for multiple mating and escaping from crowded sites. Large belly patches and several other morphological traits may assist their success in obtaining mates during dispersal. Furthermore, a relatively high relatedness (*R* = 0.06) among females suggested that this species might have rudimentary social structure. Case studies in “less” social species may provide important evidence for a better understanding of sex-biased dispersal.

## Introduction

Sex-biased dispersal, defined as the difference between sexes in dispersal rates and distances from their birth places (Lawson Handley and Perrin 2007), has profound impacts on a species' biology, such as population structure and social organization (Clobert et al. 2001). Several hypotheses have been proposed to explain the evolution of sex-biased dispersal. Competition between related males for resources or competition between related females for mates may prompt male or female to disperse more than the other sex (Greenwood 1980; Perrin and Mazalov 2000). Inbreeding avoidance can also lead to sex-biased dispersal (Greenwood 1980; Pusey 1987; Perrin and Mazalov 1999, 2000). A common theme underlying those hypotheses is that male-biased dispersal is more frequently associated with polygynous mating system, whereas female-biased dispersal is often linked to monogamous mating system (Greenwood 1980). Nevertheless, recent studies suggest that this traditional mating system hypothesis is probably oversimplified, and factors associated with sex-biased dispersal are likely multidimensional (Calsbeek 2009; Perez-Espona et al. 2010; Lane and Shine 2011).

The contribution of social interaction, such as kin cooperation, to sex-biased dispersal has recently garnered much attention (Lawson Handley and Perrin 2007). Complex social interaction may enhance the magnitude of sex-biased dispersal (e.g., Smith et al. 2011; Johnstone et al. 2012). This is particularly true for socially polygynous species, where the tremendous benefits of kin cooperation lead to female philopatry, while costs of inbreeding depression and local mate competition promote male dispersal (e.g., Perrin and Goudet 2001; Devillard et al. 2004). Many recent studies on mammals and birds tested the roles of kin cooperation on philopatry and the role of inbreeding avoidance on dispersal (e.g., Sharp et al. 2008; Meshriy et al. 2011; Arnaud et al. 2012). Nevertheless, both mammals and birds are generally considered as having well-developed kin cooperation and social structure (Pusey and Packer 1987; Sharp et al. 2008). The contribution of social interaction to sex-biased dispersal in “less social” vertebrates, such as amphibians and reptiles, has not been explored.

Lizards are excellent model systems for such tasks because they are relatively well studied and have rudimentary social properties, such as territoriality, “simple” sociality, and social displays (Wilson 1975). Many lizards are territorial and defend certain areas during their breeding season for direct fitness benefits (e.g., Stamps 1983; Fuller et al. 2005). Compared with birds and mammals, lizards exhibit less complex forms of social structure involving kin or nonkin-based aggregations that are often associated with simple forms of parental care (e.g., Reynolds et al. 2002; Huang 2006). In addition, many lizard species possess complex displays and social interaction-related morphological traits (e.g., Orrell and Jenssen 2003; Osborne 2005). For example, the threat displays in male side-blotched lizards (*Uta stansburiana*) function as advertisement of individual endurance (Brandt 2003), and the male throat colors signal individual fighting ability in Augrabies flat lizard (*Platysaurus broadleyi*; Stapley and Whiting 2006). Those social interaction-related displays and traits may reinforce each other (McElroy et al. 2007). Thus, examining the relations between sex-biased dispersal and social interaction-related morphological traits in lizards may provide important clues to the impacts of the evolution of early social structure on sex-biased dispersal.

**Figure 1:**
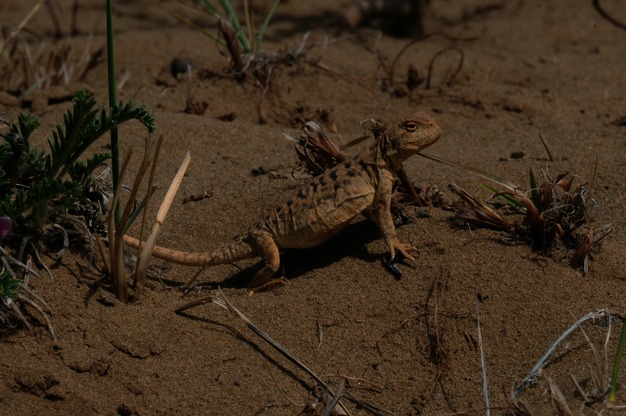
A female *Phrynocephalus vlangalii* is thermoregulating by lifting body away from substrate.

The Qinghai toad-headed agamid (*Phrynocephalus vlangalii*) is a viviparous and polygynous lizard that only occurs at high elevations of the northern Tibetan Plateau (Fig. 1; Qi et al. 2012). Both males and females are highly territorial and use complex tail displays during social interactions, such as territorial defense, male courtship, female resistance to mating, and female intrasexual interactions (Qi et al. 2011a). They also have a tail-tip badge and a belly patch which become very visible during social interactions (Qi et al. 2011a). Males use tail curling to signal body conditions and establish social ranks (Qi et al. 2011b). Our recent data show that the belly patch size is negatively correlated with male and female individual territoriality, while head size of *P. vlangalii* is positively correlated with individual bite force (Y. Qi, unpubl. data). Furthermore, the complex displays used by female *P. vlangalii* and their high levels of intrasexual aggression are unique among lizards. Compared with other well-studied lizards, such as *Uma exsul* and *Anolis nebulosus* (Carpenter 1967; Jenssen 1971), females of *P. vlangalii* use their displays during social interactions much more frequently. Such biological traits make the Qinghai toad-headed agamid an excellent study system to explore the potential contribution of social interaction to sex-biased dispersal.

We examined sex-biased dispersal in the Qinghai toad-headed agamid and its potential association with social interaction. Specifically, we aim at (1) detecting sex-biased dispersal using population genetic methods, and (2) comparing social interaction-related morphological traits among dispersers and residents. As *P. vlangalii* displays complex social interactions among females and has a polygynous mating system, we predict that females are highly philopatric and males disperse more frequently and/or with greater distance (male-biased dispersal). Consequentially, males should have less population structure (e.g., male *F*_ST_ < female *F*_ST_) and a higher dispersal rate. Furthermore, social interaction-related morphological traits, such as head size, belly patch size, and tail size, are likely different between residents and dispersers, and such differences likely vary between the two sexes. By examining these traits, we can estimate the importance of social interaction for determining sex-biased dispersal.

## Materials and Methods

### Sampling

**Figure 2:**
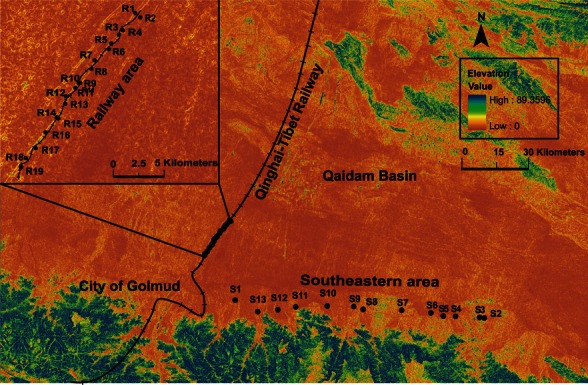
Sampling sites from the Qaidam Basin. Sites R1–R19 are nearby the Qinghai-Tibet Railway. Sites S1–S13 are from the Southeastern area, approximately 20 km away from the Railway area.

Tissue samples from 345 individuals of *P. vlangalii* were collected from the Qaidam Basin. Most samples (*n* = 219) were collected in 2010 and additional samples (*n* = 126) from the same areas were added to increase our statistical power in 2011. All sampling sites were from two areas; one was along a 20-km stretch of the Qinghai-Tibet Railway north of the city of Golmud (Fig. 2; R1–R19), and the other was located further southeast of the Railway sampling area (Fig. 2; S1–S13). There was an approximately 20 km gap between the two sampling areas, where lizards were rare. In the Railway area, all sampling sites were close to each other (0.5–2.5 km) along the railway (Fig. 2), and a total of 240 samples including 80 males and 160 females were collected from these sites. The population density at sites R15 and R12 was much higher than at the other sites, based on the observed number of burrows and number of samples that we were able to collect (Appendix S1). In the Southeastern sampling area, the sampling sites were further apart (3–15 km; Fig. 2), and a total of 105 samples including 28 males and 77 females were collected. Because of the well-spread nature of these lizards, we sampled many sites, but only collected a few individuals from the majority of sites. As our sampling effort was similar for each site, the number of samples from each site should approximate the population density of the site. Detailed information of each sampling sites is provided in Appendix S1.

Animals were captured by hand or in pitfall traps, and the sex of individuals was determined by checking for the presence of hemipenes. All individuals were euthanized in the field. Liver tissues were collected and preserved in 95% ethanol. All voucher specimens are stored in the herpetological collection of the Chengdu Institute of Biology, Chinese Academy of Sciences.

### Laboratory protocols

Genomic DNA was extracted from liver tissues using TIANamp Genomic DNA kits (Tiangen Biotech, Beijing, China) following the manufacturer's recommendation. Genotypes were determined using nine polymorphic microsatellite DNA loci (Phr27, Phr75, Phr79, Phr63, Phr160, Pvms12, Pvms18, Pvms35, and Pvms38). We used the primers developed by Urquhart et al. (2005) and Zhan and Fu (2009), and the forward primers were labeled with one of FAM, TAMRA, or HEX fluorescence dyes. All PCRs were conducted with individual pairs of primers in 25 μL reaction systems. The PCR cocktail included 20–50 ng of template DNA, 1 U *Taq* DNA polymerase (TaKaRa, Takara Bio inc., Otsu, Japan), 2 mmol/L MgCl_2_, 0.2 mmol/L of each dNTP, and 10 pmol of each primer in 1× PCR buffer (TaKaRa). PCRs were conducted with the following parameters: 5 min for initial denaturation at 95°C, followed by 30 cycles of 94°C denaturing for 30 sec, 58°C annealing for 30 sec, 72°C extension for 30 sec, and a final additional extension step at 72°C for 5 min. PCR products with different fluorescence labels were then pooled and genotyped on an ABI 3730 genetic analyser (Applied Biosystems, Foster, CA). All electropherograms were visually inspected and analyzed using genemarker version 1.6 (SoftGenetics, State College, PA). All samples were genotyped at least twice until all inconsistent results were resolved.

### Population genetic analysis

All loci were checked for null alleles with micro-checker version 2.2.3 (Van Oosterhout et al. 2004). Only sites with sample size equal or greater than five were included in null alleles detection (*N*_*site*_ = 21). Genetic diversity in the two sampling areas (Railway and Southeast) was assessed with three indices. Number of alleles (*A*), observed heterozygosity (*H*_o_), and expected heterozygosity (*H*_e_) were calculated using genepop version 4.1.3 (Rousset 2008). Deviation from Hardy–Weinberg equilibrium and linkage equilibrium in each sampling area was also analyzed with genepop.

We assessed population genetic structure of males and females separately, and used both individual genotype-based assignment tests and allele frequency-based analyses. Individual-based assignment tests are perhaps the most suitable analytical methods in our case. These tests do not require predefined population boundaries (Manel et al. 2005), which is very difficult for these lizards due to their well-spread nature. In addition, these methods do not assume that populations are at equilibrium (Manel et al. 2005). Our sampling strategy, which is “many sites but few individual from each site,” is also well suited for these tests. Consequently, we employed several assignment tests including genetic clustering, assignment index correlation, and first-generation migrant identification. We used allele frequency-based analyses as supplementary and for comparison. An analysis of molecular variance (AMOVA) and estimation of *F*_st_ as well as several other population genetic indices were conducted. Only sites with sample sizes equal or greater than five were included in this set of analyses.

To assess genetic cohesion or clustering of each sex, we use a Bayesian inference with the program structure version 2.3.1 (Pritchard et al. 2009). structure estimates number of naturally occurring genetic clusters (*K*) in the samples by optimizing Hardy–Weinberg equilibrium and minimizing linkage disequilibrium within each cluster (Pritchard et al. 2000). Assuming an admixture model with independent allele frequencies, *K* values between 1 and 6 were examined. We ran 100 independent iterations for each *K*, and each run started with 300,000 burn-in iterations and continued for 100,000 post burn-in iterations. We used successive changes of LnP(D) values, Evanno's Δ*K*, and individual assignment probabilities to assess the best value of *K* (Evanno et al. 2005). Populations with male-biased dispersal should have fewer genetic clusters among males than those among females.

Number of first-generation migrants was estimated for males and females separately, using program geneclass2 version 2.0.h (Piry et al. 2004). First-generation migrants were defined as individuals that were born at a site other than the one in which they were collected. We used Lh as the statistical criterion for likelihood computation because not all potential source sites were sampled (Paetkau et al. 2004). To determine the critical value of the test statistic at α = 0.01 level, the Bayesian method of Rannala and Mountain (1997) was used in combination with the Monte Carlo resampling algorithm of Paetkau et al. (2004). We also identified the source sites of each migrant according to their assignment results. The geographic distance between the sampling sites and source sites for each migrant was estimated using ArcMap.

Favre et al.'s (1997) mean assignment index correlation (mAIc) was also used to assess sex-bias in dispersal with the program genalex version 6.4.1 (Peakall and Smouse 2006). Negative mAIc values indicate higher than expected frequency of rare genotypes in a population, suggesting high frequency of dispersal. This method has the advantage of allowing each population or subpopulation to be tested independently, hence is able to detect dispersal bias at different geographical scales. Two spatial levels were tested. We first calculated male and female mAIc values for the Railway sampling area and the Southeastern area. The same values were also estimated for the four sites that had sample sizes greater than 25 (R12, R15, S1, and S5; Appendix S1). Two of them were from the Railway area and the other two were from the Southeastern area (Appendix S1). In a species with male-biased dispersal, we expected that mAIc for males would be negative and mAIc for females would be positive. The significance of these values was assessed with a Mann–Whitney U test as implemented in genalex.

A locus by locus AMOVA was conducted to determine the distribution of population genetic structure across various hierarchical levels using arlequin version 3.5 (Excoffier and Lischer 2011). Permutation tests (10,000 replicates) were carried out at four hierarchical levels: among sampling areas (=group; Railway vs. Southeastern areas); within areas among sites; among individuals within sites; and within individuals. Males and females were analyzed separately.

Several population genetic indices, including average expected heterozygosity within site (=gene diversity; *H*_S_), global *F*_ST_, *F*_IS_, and mean relatedness (*R*), were calculated separately for males and females using fstat version 2.9.3 (Goudet 1995). *R* is defined as mean relatedness among lizards of the same sex and estimated as *R* = 2*F*_ST_/(1 + *F*_IT_) where *F*_IT_ is the inbreeding coefficient of individuals relative to random mating (Queller and Goodnight 1989). Statistical significance for these indices was determined by 10,000 randomizations. All those tests require large sample sizes from each site, so we only examined the four sites with samples sizes greater than 25 (R12, R15, S1, and S5; Appendix S1). A two-sided *t*-statistic was used to test for significant difference. The more dispersing sex should have a higher *H*_S_, a lower *F*_ST_, a higher *F*_IS_, and a smaller *R* (Goudet et al. 2002; Prugnolle and De Meeus 2002).

### Social interaction-related morphological traits

To examine potential links between dispersal and social interactions, we measured eight morphological traits that are known to be associated with social interactions (Qi et al. 2011b). These traits included snout-vent length (SVL), head length (HL), head width (HW), head depth (HD), belly patch length (BPL; the maximum distance of the belly patch in parallel with the body), belly patch width (BPW; the maximum distance of the belly patch perpendicular to the body), tail length (TL), and tail-tip length (TTL). These lizards have black (male) or orange (female) tail tips, and TTL is the length of the colored section. All traits were measured to the nearest 0.01 mm with calipers.

The SVL between migrants and residents, which were identified by genetic analysis, was compared with a one-way analysis of variance (ANOVA). The other seven traits, HL, HW, HD, BPL, BPW, TL, and TTL were compared with an analysis of covariance (ANCOVA), while SVL was controlled for. All analyses were carried out using R version 2.14 (R Development Core Team 2011). Males and females were assessed separately. The distribution normality of each variable was evaluated using the Shapiro–Wilk normality test. Homogeneity of variance was evaluated using Bartlett's test. When necessary, variables were log transformed to meet the requirements of parametric tests. Female residents had a much larger sample size (*n* = 136) than migrants (*n* = 25), which produced a bimodal distribution. Subsequently, we reduced the sample size to 25 by randomly sampling female residents to obtain a normal distribution. This process was repeated three times. To remove the potential influence of annual morphological variation, we only measured samples collected in 2010, thus three male migrants and six female migrants from the year 2011 were excluded in morphological comparisons.

## Results

### Genetic diversity

We did not detect any null alleles. Number of alleles, observed heterozygosity (*H*_o_), and expected heterozygosity (*H*_e_) are presented in Table 1. The number of alleles at each locus ranged from 13 to 35, and *H*_e_ ranged from 0.79 to 0.94. The overall genetic diversity was relatively high, and this is consistent with previous studies on this species (Wang et al. 2009). Seven loci in the Railway sampling area (sample pooled), and six loci in the Southeastern area showed significant departure from Hardy–Weinberg equilibrium after Bonferroni correction (Table 1). Two (of the 36) pairs of loci were in significant linkage disequilibrium; however, no pair of loci was consistently in linkage disequilibrium across all sampling sites. Thus, we assumed that these loci were not in physical linkage. These results were not surprising because samples were pooled from multiple sites. These departures from equilibrium were likely results of the Wahlund's effect (Hartl 2000).

**Table 1:** Genetic diversity of the Qinghai toad-headed agamid *Phrynocephalus vlangalii* at nine microsatellite DNA loci

Location	Total number	Female	Male	Phr27	Phr75	Phr160	PVMS38	PVMS35	PVMS12	PVMS18	Phr79	Phr63
Railway												
*A*	239	159	80	25	35	18	32	20	23	26	24	16
*H*_o_				0.7314	0.8471	0.7718	0.8595	0.6240	0.5372	0.6529	0.7893	0.8306
*H*_e_				0.9233	0.9253	0.8271	0.8950	0.8911	0.7932	0.8845	0.9097	0.8784
*P*_HW_				**<0.0005**	**<0.0005**	**<0.0005**	0.044	**<0.0005**	**<0.0005**	**<0.0005**	**<0.0005**	0.224
Southeastern												
*A*	106	78	28	30	34	13	26	20	23	23	20	18
*H*_o_				0.7453	0.7642	0.7170	0.8208	0.8000	0.6604	0.7736	0.7830	0.8774
*H*_e_				0.9444	0.9366	0.7949	0.9322	0.9205	0.8979	0.9265	0.9203	0.9003
*P*_HW_				**<0.0005**	**<0.0005**	0.028	0.014	**<0.0005**	**<0.0005**	**<0.0005**	**<0.0005**	0.450

*A*, number of alleles; *H*_o_, observed heterozygosity; *H*_e_, expected heterozygosity; *P*_HW_, *P*-value of Hardy–Weinberg equilibrium test. Bold indicates significance after sequential Bonferroni correction at 0.01 level.

### Population genetic structure among male and female *P. vlangalii*

**Figure 3:**
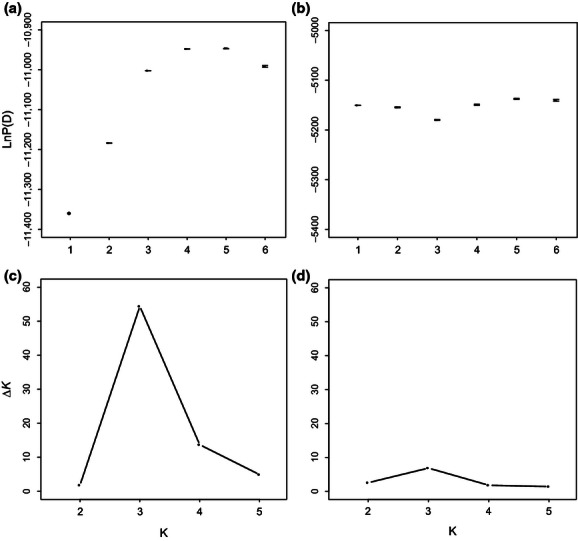
Average likelihood values and Δ*K* for *K* = 1–6 from the structure analysis. LnP(D) values are average of the likelihood values from 100 independent runs. Δ*K* was calculated following the procedures suggested by Evanno et al. (2005). (A and C) Females; (B and D) males.

**Figure 4:**
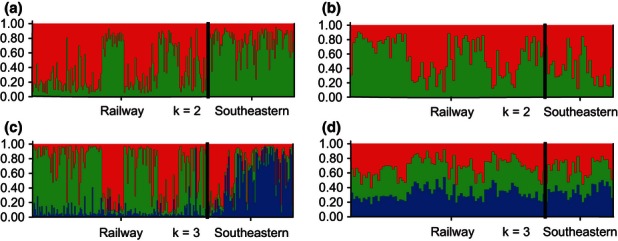
Individual assignment probability bar plots for *K* = 2–3 from the structure analysis. Each vertical bar represents one individual. Individuals from the Railway area are arranged in the order from north to south, and individuals from the Southeastern area are arranged in the order from west to east. (A and C) Females; (B and D) males.

Figure 3 presents the LnP(D) values for each *K* and Figure 4 presents the individual assignment probabilities from the structure assignment tests. These tests inferred three genetic clusters among females, but only one cluster among males. For females, the successive changes of LnP(D) showed a significant increase when *K* changed from 2 to 3, and the increase in LnP(D) was minimal with subsequent changes of *K* (Fig. 3A). Concordantly, Δ*K* revealed a clear peak at *K* = 3 (Fig. 3C). Furthermore, approximately half of the females received assignment probabilities greater than 90%, and three quarters of individuals had probabilities greater than 80% when *K* = 3 (Fig. 4C). For males, we did not find a significant increase in LnP(D) with changes in *K* (Fig. 3B), and Δ*K* did not show any clear peak (Fig. 3D). In addition, a high proportion (95%) of males received assignment probabilities less than 80% when *K* = 2 or 3 (Fig. 4B and D), suggesting one cluster was the best fit for males. The larger number of genetic clusters among females than among males is consistent with male-biased dispersal.

geneclass2 identified 23 males to be first-generation migrants and 75 males to be residents of a total of 108 samples. The status of the remaining 10 males was uncertain. Of the 237 females, we detected 31 migrants, 193 residents, and 13 with uncertain status. The proportion of male migrants to residents (23:75) was significantly higher than that of females (31:193) (Pearson's chi-squared test, χ^2^ = 3.87, df = 1, *P* = 0.04). Furthermore, the majority of male migrants (18/23) were from three high-density sites (R12, R15, S1), while only a small proportion of female migrants (7/31) originated from high-density sites (R12, R15, S1, S5). A small proportion of male migrants (4/23) dispersed between neighboring populations (<1 km), whereas the majority of them (19/23) performed long-distance (>1 km) movements, of which seven were found crossing between the Railway and Southeastern areas (>20 km). Similarly, a small number of female migrants (3/31) dispersed between neighboring populations (<1 km), and 21 were within the Railway or Southeastern area (>1 km) while seven moved between the two sampling areas. Average dispersal distances and frequencies of dispersal events at different distances did not differ significantly between male and female migrants (Wilcoxon rank sum test, *P* = 0.051). However, considering the much larger sample size of females, the proportion of long-distance dispersal in males (7/108) was much higher than in females (7/237). This is consistent with the male-biased dispersal hypothesis.

**Figure 5:**
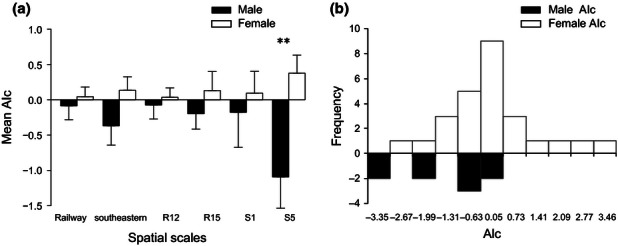
(A) Comparisons of mean assignment index correlation (AIc) values between males and females in the two sampling areas (Railway and Southeastern) and four sites (R12, R15, S1, and S5). **Significant at *P* < 0.01. (B) Detailed AIc distribution of site S5.

Mean AIc values were negative for males and positive for females in the two sampling areas and in the four sites examined (Fig. 5), suggesting that the rare genotypes were much easier to find among males than females. At both spatial scales, area and site, the patterns were the same and were consistent with male-biased dispersal. In one southeastern sample site (S5), the difference in mean AIc value between males (*n* = 9) and females (*n* = 26) was significant (*Z* = −2.72, *P* = 0.007), while in other areas and sites, the difference was not significant (*P* > 0.05).

AMOVA revealed different population genetic structure between the two sexes (Table 2). There was clearly a small (2%), but significant amount of structure between the Railway and Southeastern sampling areas among females (*P* < 0.001, Table 2). For males, however, there was no significant structure between the two areas (*P* = 0.051, Table 2). This result suggested that males might have more frequent long-distance dispersal (>20 km) between the two areas, which is consistent with our findings from the geneclass2 analysis.

**Table 2:** Results of the analysis of molecular variance

Source of variation	Sum of square	Variance components	Percentage variation
Male			
Among groups	7.82	0.04	0.87*
Among sites within groups	12.74	0.06	1.46**
Among individuals within sites	276.39	0.42	10.47**
Within individuals	237.00	3.53	87.19**
Total	533.96	4.06	
Female			
Among groups	24.44	0.09	2.15**
Among sites within groups	107.05	0.12	2.99**
Among individuals within sites	826.73	0.52	12.60**
Within individuals	690.00	3.37	82.26**
Total	1648.22	4.09	

Two groups are defined as (1) all sites from the Railway area and (2) all sites from the Southeastern area. Only sites with sample sizes greater than five were included in this analysis. Average over nine loci; number of permutations = 10,000; allowed level of missing data = 0.05.

* *P* = 0.0506,

** *P* < 0.001.

All estimated population genetic indices are presented in Table 3. Males display significantly lower population differentiation (*F*_ST_) and lower relatedness (*R*) than females, all being consistent with male-biased dispersal. The gene diversity (*H*_S_) and *F*_IS_ is compatible with male-biased dispersal, but does not reveal significant differences between the two sexes (Table 3).

**Table 3:** Differences between male and female of the Qinghai toad-headed agamid *Phrynocephalus vlangalii* in gene diversity (*H*_S_), *F*_ST_, *F*_IS_, mean relatedness (*R*)

	*H*_S_	*F*_ST_	*F*_IS_	*R*
Female	0.872	0.035	0.097	0.064
Male	0.881	0.019	0.107	0.035
*P*-value	0.20	**0.04**	0.65	**0.04**

*P*-values are from two-tailed *t*-tests; bold indicates significance at *P* < 0.05.

### Social interaction-related morphological traits

Migrants and residents possessed different morphological traits in both males and females, and often the differences were in opposite direction between males and females (Table 4). For males, migrants (*n* = 20) had significantly larger HL, HD, and HW than residents (*n* = 23). The SVL, BPL, BPW, TL of male migrants were also larger than those of residents, although the differences were not statistically significant (*P* > 0.05). For females, migrants (*n* = 25) had significantly smaller HL, HW, and TL than residents (*n* = 25), and the SVL, HD, BPL, BPW, and TTL of migrants were also smaller than those of residents, but not significant (*P* > 0.05).

**Table 4:** Comparisons of eight metric morphological traits between migrants and residents

	Males			Females		
		
	Migrants (*n* = 20)	Residents (*n* = 23)	Difference	Migrants (*n* = 25)	Residents (*n* = 25)	Difference
SVL	51.28 ± 0.80	49.72 ± 0.81	*F*_1,41_ = 1.86, *P* = 0.18	49.32 ± 1.29	52.13 ± 1.33	*F*_1,47_ *=* 2.26, *P* = 0.14
HL	16.07 ± 0.24	15.18 ± 0. 30	***F***_**1,39**_ ***=*** **15.46**, ***P*** **<** **0.001**	14.89 ± 0.31	15.42 ± 0.32	***F***_**1,46**_ ***=*** **9.32**, ***P*** **=** **0.004**
HW	13.73 ± 0.32	12.66 ± 0.27	***F***_**1,39**_ ***=*** **16.71**, ***P*** **<** **0.001**	12.85 ± 0.37	13.64 ± 0.49	***F***_**1,46**_ ***=*** **4.67**, ***P*** **=** **0.04**
HD	8.21 ± 0.16	7.86 ± 0.19	***F***_**1,39**_ ***=*** **6.67**, ***P*** **=** **0.01**	7.79 ± 0.19	8.07 ± 0.21	*F*_1,46_ *=* 2.07, *P* = 0.16
BPL	22.01 ± 0.57	20.87 ± 0.80	*F*_1,38_ *=* 1.54, *P* = 0.22	19.72 ± 1.32	21.89 ± 1.31	*F*_1,46_ *=* 0.38, *P* = 0.54
BPW	7.66 ± 0.28	6.99 ± 0.29	*F*_1,38_ *=* 2.96, *P* = 0.09	4.89 ± 0.45	5.72 ± 0.44	*F*_1,46_ *=* 0.001, *P* = 0.97
TL	55.78 ± 0.99	54.36 ± 1.16	*F*_1,39_ *=* 1.55, *P* = 0.22	49.52 ± 1.12	51.78 ± 1.40	***F***_**1,46**_ ***=*** **10.43**, ***P*** **=** **0.002**
TTL	19.94 ± 0.60	20.27 ± 0.78	*F*_1,36_ *=* 0.11, *P* = 0.74	15.93 ± 0.64	16.44 ± 0.77	*F*_1,46_ *=* 2.35, *P* = 0.13

All measurements are in mm. Significant differences between SVLs are determined by one-way ANOVA, and differences between other traits are determined by ANCOVA while SVL is controlled for. Bold indicates significance at *P* < 0.05. SVL, snout-vent length; HL, Head length; HW, Head width; HD, Head depth; BPL, Belly patch length; BPW, Belly patch width; TL, Tail length; TTL, Tail tip length; ANOVA, analysis of variance; ANCOVA, analysis of covariance.

## Discussion

We found strong evidence for male-biased dispersal in *P. vlangalii*. Results from both individual-based assignment tests and frequency-based analyses were consistent with male-biased dispersal. More importantly perhaps, we detected an opposite trend in variation in social interaction-related morphological traits between males and females. While male residents have smaller heads than male migrants, female residents have larger heads and longer tails than migrants. This observation suggests that social interaction may play a significant role in determining the dispersal patterns.

### Evidence for male-biased dispersal in *P. vlangalii*

Our data are consistent with the prediction of a male-biased dispersal hypothesis. First, assignment tests detected more genetic clusters among females than males (Figs. 3 and 4), and identified a higher proportion of first-generation migrants among males than among females. Second, the mean *AIc* values were negative for males and positive for females (Fig. 5). Third, allele frequency-based analysis revealed a higher *H*_S_, a lower *F*_ST_, a higher *F*_IS_, and a smaller *R* among males than those among females. All these results are consistent with the male being the higher dispersal sex (Prugnolle and De Meeus 2002).

Our finding is not surprising considering that most known sex-biased dispersal cases in lizards are male-biased. Although studies of sex-biased dispersal in non-mammal/bird organisms are relatively few, several lizard species have been reported to exhibit male-biased dispersal, including *Lacerta vivipara* (Clobert et al. 1994), *U. stansburiana* (Doughty et al. 1994), *Lacerta agilis* (Olsson et al. 1996), *Egernia stockesii* (Gardner et al. 2001), *Sceloporus occidentalis* (Massot et al. 2003), *Egernia frerei* (Fuller et al. 2005), *Anolis roquet* (Johansson et al. 2008), *Chlamydosaurus kingii* (Ujvari et al. 2008), *Phrynocephalus przewalskii* (Urquhart et al. 2009), and *Eulamprus leuraensis* (Dubey and Shine 2010). There is only one reported case of female-biased dispersal in *Niveoscincus microlepidotus*, which was attributed to females having strict selection on oviposition sites (Olsson and Shine 2003). Lizard species with male-biased dispersal have been reported to have polygynous, monogamous, and flexible mating systems (Gardner et al. 2001; Massot et al. 2003; Chapple and Keogh 2005).

Male and female dispersers in *P. vlangalii* share some characteristics, but differ in others. For example, in terms of dispersal distance preference, dispersers of both sexes appear to favor medium migration distance between 1 and 20 km. On the other hand, a large proportion of male migrants (18/23) are from high-density populations. Considering the total number of males from these populations (58) and total number of males in our study (108), the proportion is much higher than a random draw, and therefore, cannot be due to chance alone. The majority of female migrants (24/31) are from low-density populations, and the proportion is also higher than that by a random draw. This is likely related to the different social behavior and selection pressures between sexes (Clutton-Brock 2007).

Sex-biased dispersal patterns of *P. vlangalii* appear to be similar at the two spatial scales that we examined. Our spatial scale ranged from 0.5 to >20 km; it covered most hierarchical levels at which dispersal was thought to occur in most vertebrates (Koenig et al. 1996). This appears to be the rule in most organisms (Lawson Handley and Perrin 2007). The only known exception to this rule is the greater white-toothed shrew (Fontanillas et al. 2004), in which the dispersal is female-biased at a local scale, but male-biased at a large scale.

### The cost-benefit dynamic and the importance of social interaction

To disperse or to remain on a natal site is fundamentally the consequence of a cost-benefit dynamic (Clobert et al. 1994; Lawson Handley and Perrin 2007). In addition to resource and inbreeding-related costs and benefits, social interaction in *P. vlangalii* likely contributes to the dynamic. This inference is based on the observed variation in social interaction-related morphological traits between dispersers and residents. The fact that these variations are different between males and females suggests that the cost-benefit dynamics are also different between the two sexes.

The benefits of philopatry for female *P. vlangalii* are probably large. Like many other polygynous species, females of *P. vlangalii* invest more heavily than males, and tend to stay near their familiar areas so that they may acquire enough resources for their offspring (Gregory et al. 1987). *Phrynocephalus vlangalii* is viviparous, and viviparity further increased the maternal investment. In addition, females need to experience 2 months gestation time (Y. Qi, pers. obs.) before parturition, which makes dispersal dangerous and costly (Shine 1980). Other advantages may include intimate knowledge of local refuges, risk factors, and prey availability (Johansson et al. 2008; Dubey and Shine 2010). These benefits for females are well studied (e.g., Lawson Handley and Perrin 2007) and mostly in line with the mating system hypothesis, and *P. vlangalii* is no exception.

Social interaction among *P. vlangalii* may further increase the benefits of female philopatry. Although we do not have direct evidence to suggest social cooperation among females, the complex social interactions with conspecifics and frequently used displays may help females defend territories and prevent social conflicts from escalating into fights, and thereby reduce the costs of staying and promote female philopatry (Qi et al. 2011b). Females were also observed to engage in frequent burrow defense behavior before giving birth and then share their burrows with offspring after giving birth (Qi et al. 2012). Morphological data suggest that female residents of *P. vlangalii* are likely dominant individuals, and subordinates tend to disperse. Resident females of *P. vlangalii* have larger heads than dispersers; head size is an important predictor of individual bite force and has been suggested as an important indicator of social dominance in lizards (KratochvÍl and Frynta 2002). Furthermore, we also found that residents have larger and more obvious social interaction-related morphological traits, such as tail length. Tail length is likely an indicator of female body condition, and is also commonly used in tail display signals (Qi et al. 2011a). The larger and more obvious traits in residents suggest that social interaction may be helpful and important for females' survival in a local community. Few female dispersers are from high-density populations (7/31); this may suggest that social interactions in high-density populations are more frequent and benefits from such interactions are also higher.

For male *P. vlangalii*, the cost and benefit dynamic is likely quite different from that of females. Contrary to females, male dispersers are dominants with larger heads, suggesting dispersal may confer certain selective advantages. We hypothesize several potential benefits. First, dispersal may provide males with more mating opportunities and this is consistent with its polygynous mating system. Females of *P. vlangalii* do not form aggregations, which are probably related to their strong territoriality and aggression. Males use coercion as their primary mating strategy. These characteristics suggest that dispersal can be an effective male strategy to acquire extra mating opportunities. Second, dispersal may enable males to escape from crowded populations and to colonize new territories (Matthysen 2005). This is congruent with our observation that a high proportion (18/23) of male dispersers were from high-density sites. Third, male dispersers possess larger belly patches, which are involved in social communication and are correlated with individual territoriality (Y. Qi, unpubl. data). This suggests that social interactions may be used for rival assessment during dispersal and to reduce the potential costs associated with dispersal.

Despite dispersal being male-biased in *P. vlangalii*, only a small proportion of the population were first-generation migrants, and the majority (males 69%, females 81%) were residents in both sexes, indicating strong overall costs associated with dispersal. This is drastically different from “true” social species, such as lions in which all males leave their natal prides, while most females remain (Pusey and Packer 1987). Furthermore, females of *P. vlangalii* have a within-site mean relatedness of 0.06, while males have a mean relatedness of 0.03. These are smaller than “true” social species (e.g., lions: *R*_*female*_ > 0.5, Packer and Pusey 1987; lizard *Egernia stokesii*: *R*_*female*_ = 0.138, Gardner et al. 2001), but larger than other nonsocial lizards (e.g., *Tiliqua rugosa*, *R*_*female*_ = −0.021, *R*_*male*_ = −0.022; Bull and Cooper 1999). *Phrynocephalus vlangalii* likely possess a rudimentary social structure. Considering that the majority of both males and females are residents, there is a potential risk for inbreeding. We suggest that females may have the ability to recognize kin by signal or other means and actively choose unrelated partners in a similar way as in several other lizard species (*Egernia striolata*, Bull and Cooper 1999; *E. stokesii*, Gardner et al. 2001; *T. rugosa*, Bull et al. 2001). More research is needed to further understand inbreeding avoidance mechanisms in *P. vlangalii* and their relation to sex-biased dispersal.

### Concluding remarks

The toad-headed agamid *P. vlangalii* is strongly biased toward male dispersal. This is largely consistent with its polygynous mating system and the observed general trend in lizards. Furthermore, complex social interaction, coupled with viviparity, may enhance female philopatry in this species, while promoting male dispersal. The large percentage of residents in both sexes and the relative high within-site relatedness may suggest existing rudimentary social structure and potential kin recognition ability.

Sex-biased dispersal is probably common across animal groups and the underline mechanisms are clearly multidimensional. Many mechanisms, such as mating system and social complexity, may work together and hence are difficult to tease apart. More case studies, particularly among non-mammal/bird species, are needed to generate and to test widely applicable hypotheses.

## Data Accessibility

All raw microsatellite DNA genotype data and morphological measurement data are available as online supporting information.

## Supporting Information

**Appendix S1.** Descriptions of sampling sites of the Qinghai toad-headed agamid *Phrynocephalus vlangalii*.

**Appendix S2:** Raw microsatellite DNA genotype data.

**Appendix S3:** Raw morphological measurement data.
